# Supplementary material for: Epidemiological Study of Multiple Zoonotic Mosquito-Borne Alphaviruses in Horses in Queensland, Australia (2018–2020)
Source: Viruses. 2022 Aug 23;14(9):1846. doi: 10.3390/v14091846 (PMC9504300; doi:10.3390/v14091846)
Supplement: Supplementary file 1 [file viruses-14-01846-s001.zip › viruses-1875448-supplementary.pdf]

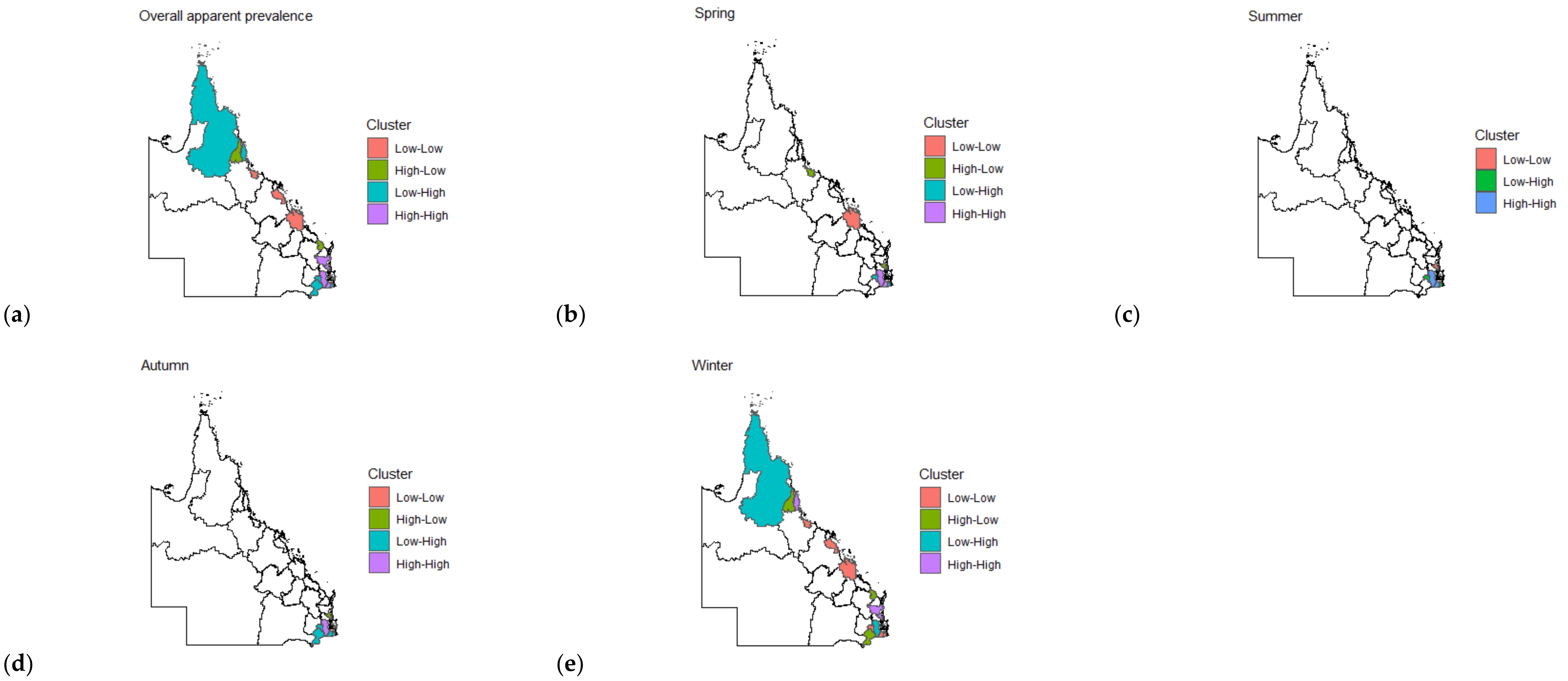


**Figure S1.** Local spatial clustering of RRV seropositivity in QLD categorized by entire dataset (**a**) and by seasons (**b**–**e**): spring (**b**), summer (**c**), autumn (**d**), winter (**e**).

**Table S1.** Stratified analysis of risk factors categorized by sample populations.

| | | QRIC | | UQ | |
|---|---|---|---|---|---|
| **Risk Factors** | **Level** | **RRV sero-positive % (N)** | **RRV sero-negative % (N)** | **RRV sero-positive % (N)** | **RRV sero-negative % (N)** |
| **Sex** | Female | 36.2 (71) | 63.8 (125) | 82.6 (57) | 17.4 (12) |
| | Male | 43.7 (139) | 56.3 (179) | 89.7 (35) | 10.3 (4) |
| | P-value* | 0.097 | | 0.404 | |
| **Age at sampling (years)** | 2 – 6 | 38.9 (174) | 61.1 (273) | 77.8 (21) | 22.2 (6) |
| | > 6 | 53.7 (36) | 46.3 (31) | 87.7 (71) | 12.3 (10) |
| | P-value* | 0.024 | | 0.222 | |
| **Season** | Spring | 39.6 (38) | 60.4 (58) | 82.6 (57) | 17.4 (12) |
| | Summer | 41.3 (50) | 58.7 (71) | 91.7 (11) | 8.3 (1) |
| | Autumn | 49.0 (47) | 51.0 (49) | 83.3 (15) | 16.7 (3) |
| | Winter | 37.3 (75) | 62.7 (126) | 100 (9) | 0 (0) |
| | P-value* | 0.295 | | 0.646 | |

* Fisher's exact test.
